# The prognostic value of epidermal growth factor receptor mRNA expression in primary ovarian cancer.

**DOI:** 10.1038/bjc.1996.53

**Published:** 1996-02

**Authors:** J. M. Bartlett, S. P. Langdon, B. J. Simpson, M. Stewart, D. Katsaros, P. Sismondi, S. Love, W. N. Scott, A. R. Williams, A. M. Lessells, K. G. Macleod, J. F. Smyth, W. R. Miller

**Affiliations:** Imperial Cancer Research Fund Medical Oncology Unit, Western General Hospital, Edinburgh, UK.

## Abstract

**Images:**


British Journal of Cancer (1996) 73, 301-306
© 1996 Stockton Press All rights reserved 0007-0920/96 $12.00

### The prognostic value of epidermal growth factor receptor mRNA expression in primary ovarian cancer

JMS Bartlett[1,*], SP Langdon[1], BJB Simpson[1], M Stewart[1], D Katsaros[2], P Sismondi[2], S Love[3], WN Scott[1], ARW Williams[4], AM Lessells[5], KG Macleod[1], JF Smyth[1] and WR Miller[1]

[1]*Imperial Cancer Research Fund Medical Oncology Unit, Western General Hospital, Edinburgh EH4 2XU;* [2]*Department of Gynecologic Oncology, University of Turin, Turin, Italy;* [3]*Imperial Cancer Research Fund Medical Statistics Laboratory, Lincoln's Inn Fields, London WC2A 3PX;* [4]*Department of Pathology, University of Edinburgh, Teviot Place, Edinburgh EH8 9AG;* [5]*Department of Pathology, Western General Hospital, Edinburgh EH4 2XU, UK.*

**Summary** The expression of mRNA for the epidermal growth factor (EGF) receptor, EGF and transforming growth factor α (TGF-α) was determined in 76 malignant, six borderline and 15 benign primary ovarian tumours using the reverse transcriptase-polymerase chain reaction and related to clinical and pathological parameters. Of the malignant tumours, 70% (53/76) expressed EGF receptor mRNA, 31% (23/75) expressed EGF mRNA and 35% (26/75) expressed TGF-α mRNA. For the borderline tumours, four of six (67%) expressed EGF receptor mRNA, 1/6 (17%) expressed TGF-α mRNA and none expressed EGF mRNA. Finally, 33% (5/15) of the benign tumours expressed EGF receptor mRNA, whereas 40% (6/15) expressed EGF mRNA and 7% (1/15) expressed TGF-α mRNA. The presence of the EGF receptor in malignant tumours was associated with that of TGF-α ($P=0.0015$) but not with EGF ($P=1.00$), whereas there was no relationship between the presence of EGF and TGF-α ($P=1.00$). EGF receptor mRNA expression was significantly and positively associated with serous histology ($P=0.006$) but not with stage or grade. Neither EGF nor TGF-α showed any link with histological subtype or stage. The survival of patients with malignant tumours possessing EGF receptor mRNA was significantly reduced compared with that of patients whose tumours were negative ($P=0.030$ for all malignant tumours; $P=0.007$ for malignant epithelial tumours only). In contrast, neither the expression of TGF-α nor EGF was related to survival. These data suggest that the presence of EGF receptor mRNA is associated with poor prognosis in primary ovarian cancer.

**Keywords:** epidermal growth factor receptor; ovarian cancer; reverse transcriptase-polymerase chain reaction

Ovarian cancer is the most fatal gynaecological cancer in the UK, resulting in approximately 4000 deaths per annum (La Vecchia *et al.*, 1992). The biological factors that regulate the growth of this disease are poorly defined, although evidence is accumulating that members of the epidermal growth factor (EGF) family are implicated in the regulation of cell proliferation in ovarian tumours. In epithelial cells, both EGF and its structural homologue, transforming growth factor α (TGF-α) stimulate proliferation. TGF-α is a 50 amino acid protein that binds to the EGF receptor, and produces biological effects barely distinguishable from those of EGF. Although TGF-α was named for its ability to transform certain types of cells in culture, it is produced by normal as well as malignant cells (Berchuck and Bast, 1993). The EGF receptor is a 170 kDa glycosylated membrane-spanning protein that has served as a prototype for studies of tyrosine kinase receptors. Both EGF and TGF-α bind to the extracellular domain of the EGF receptor with equal affinity (Berchuck and Bast, 1993). Using model systems of ovarian cancer, EGF and TGF-α have been shown to be growth stimulatory to ovarian cancer cells *in vitro* (Morishige *et al.*, 1991; Rodriguez *et al.*, 1991; Scambia *et al.*, 1991; Crew *et al.*, 1992; Zhou and Leung, 1992). Antibodies directed against either TGF-α or the EGF receptor can inhibit the proliferation of ovarian cancer cell lines that both produce TGF-α and possess the EGF receptor; this is consistent with an autocrine growth stimulation pathway (Morishige *et al.*, 1991; Jindal *et al.*, 1994). Previous studies investigating the presence of EGF receptor in ovarian tumours, using either ligand binding (Bauknecht *et al.*, 1988; Battaglia *et al.*, 1989; Morishige *et al.*, 1991; Owens *et al.*, 1991*a*; Henzen-Logmans *et al.*, 1992) immunohistochemical technology (Berchuck *et al.*, 1991; Morishige *et al.*, 1991; Henzen-Logmans *et al.*, 1992) or both (Owens *et al.*, 1992), have indicated that this receptor is commonly present with incidence rates varying from 33% to 75% (Bauknecht *et al.*, 1988, 1993; Battaglia *et al.*, 1989; Berchuck *et al.*, 1991; Morishige *et al.*, 1991; Owens *et al.*, 1991*a*; Henzen-Logmans *et al.*, 1992). The levels of EGF receptor have been claimed to be higher in malignant ovarian tumours than in benign tumours or the normal ovary (Berns *et al.*, 1992; Owens and Leake, 1993) but perhaps the strongest suggestion of a biological role for EGF-like factors derives from reports that the presence of EGF receptor protein in ovarian tumours may be related to patient prognosis (Bauknecht *et al.*, 1988; Battaglia, 1989; Foekens *et al.*, 1990; Berchuck *et al.*, 1991; Scambia *et al.*, 1992), although this has not been a universal finding (Bauknecht *et al.*, 1990; Van der Burg *et al.*, 1993).

In the present study, we have examined the mRNA expression of the EGF receptor, EGF and TGF-α using the reverse transcriptase-polymerase chain reaction (RT-PCR) in 97 ovarian tumours. The expression of these growth factors and their receptors has been related to clinical and pathological parameters to evaluate further the role of the EGF receptor and its ligands in ovarian disease.

### Patients and methods

### *Patients*

Ovarian tumour material was collected from patients undergoing surgery at the Eastern General Hospital, Edinburgh, the Royal Infirmary of Edinburgh and the University Hospital, Turin. A total of 97 patients with histologically confirmed primary ovarian tumours were included in this study. The 97 tumours included 76 malignant, six borderline and 15 benign tumours. The malignant group consisted of 35 serous, 22 endometrioid,

Correspondence: SP Langdon
*Present address: Glasgow University Department of Surgery, Level II, Queen Elizabeth Building, Glasgow Royal Infirmary, Glasgow G31 2ER, UK
Received 9 February 1995; revised 17 August 1995; accepted 11 September 1995

five mucinous, five clear-cell carcinomas, three malignant mixed mesodermal tumours and six others including a steroid cell tumour, a teratoma, a mullerian tumour, two undifferentiated tumours and one of mixed histologies. Ovarian cancer patients were staged according to the International Federation of Gynecologists and Obstetricians (FIGO) criteria (1987). The staging system defined by FIGO assumes that an adequate staging operation has been performed (Cannistra, 1993). The staging operation included collection of ascites or peritoneal washing from the pelvis, gutters and diaphragms for cytological studies; total abdominal hysterectomy plus bilateral salpingoophorectomy; infracolic omentectomy and appendectomy; selective pelvic and para-aortic lymphadenectomy and debulking of all gross disease. If obvious macroscopic tumour was not present, biopsy of any lesion suspect for tumour metastasis or any adhesion adjacent to the primary tumour; blind biopsies of bladder peritoneum and cul-de-sac, right and left paracolic gutter and pelvic side walls; biopsy or smear of right hemidiaphragm, were performed. Survival data were available for 70 of the 76 patients with malignant tumours. Of these, 44 patients received cisplatin-containing regimens, seven received chlorambucil monotherapy and one was treated with $^{32}P$ intraperitoneal radiotherapy. Eighteen received no further treatment after surgery (mainly stage Ia, grade 1 disease). No patient received therapy before surgery.

### *Tumour samples*

Tumour samples were collected at the time of primary surgery, were frozen in liquid nitrogen and stored at −180°C until used. Tumour histology and grade were assessed on paraffin-embedded sections and classified according to WHO criteria (Serov and Scully, 1973).

### *mRNA extraction*

Before RNA extraction 400 mg of tumour tissue was homogenised using a tissue dismembranator (Braun, Germany) at −20°C. Total cellular RNA was extracted from frozen tissue using the lithium chloride-urea method (Bartlett, 1992). Pelleted RNA was resuspended in diethyl pyrocarbonate-treated water (DEPC water) and the concentration assessed by measuring absorbance at 260 and 280 nm.

### *RT–PCR*

RT–PCR for EGF receptor, EGF, TGF-α and γ-actin were carried out using a Techne PHC-3 thermocycler. For the reverse transcription assay, 20 μg aliquots of total cellular RNA were reverse transcribed by incubation with 300 ng of a random hexamer oligonucleotide with 2 mM each of dATP, dTTP, dCTP and dGTP (Pharmacia, UK), 200 units of Superscript reverse transcriptase (Life Technologies, Paisley, UK) for 1 h at 42°C in a total volume of 20 μl. Reverse-transcribed RNA was stored at −20°C before analysis by PCR.

For all PCRs, 0.2–1 μl of reverse transcribed RNA was added to 100 ng of each primer in a volume of 50 μl. Before PCR, this reaction was heated to 94°C for 10 min and then cooled rapidly to 4°C. PCR reactions were performed in a final volume of 100 μl containing the following: 0.5 units of *Taq* polymerase (Promega, Southampton, UK), 1.25 μM dATP, dTTP, dCTP and dGTP (Pharmacia, UK), 100 ng of each primer, 50 mM potassium chloride, 10 mM Tris-HCl, 0.1% Triton-X and 2.5 mM magnesium chloride. Reactions were overlaid with 100 μl of paraffin oil.

The amplification reaction was carried out over 40 cycles with the following parameters: step 1, 94°C for 38 s; step 2, 50°C for 53 s; step 3, 72°C for 68 s. For the final cycle, the 72°C step was extended to 7 min to ensure that all transcripts were full length. The primers used in these reactions are listed in Table I.

PCR products were visualised after electrophoresis on polyacrylamide gels and staining with ethidium bromide. Tumours were scored as positive for EGF receptor, EGF or TGF-α when a PCR product of the correct molecular size was amplified and identified (by eye) following electrophoresis. Samples were sized using a 100 bp ladder (Gibco, UK). As an additional control, PCR of a known housekeeping gene γ-actin was performed to establish the integrity of transcribed RNA.

The identities of transcript sequences were confirmed by Southern blot analysis using specific [$^{32}$P]5′-end labelled probes (25-mers) targeted to unique sequences within the transcripts (Table I).

### *Statistics*

Differences between subgroups in contingency tables were analysed by the Fisher's exact test. Differences of survival used the graphical Kaplan–Meier method and groups were compared using the log-rank test. To assess if the mRNA variables were independently prognostic for survival Cox regression analysis was used with stage, residual disease, grade and histology included among the variables in the initial step of a backward stepwise selection procedure. The results are given as hazard ratios. A hazard ratio of 2 for a given marker indicates that the risk of death at any time for a patient with that marker is twice that of a patient without the marker, all other prognostic variables being the same (Cox, 1972).

**Table I** Oligonucleotide primers and probes used for mRNA phenotyping of EGF receptor, EGF and TGF-α in primary ovarian tumours

| *mRNA* | *Primer/probe* | *Sequences* | *Position*[a] | *Transcript size* |
|---|---|---|---|---|
| EGF receptor | Sense primer | 5′-ACCTGCGTGAAGAAGTGTCC-3′ | 442–461 | 516 |
| | Antisense primer | 5′-CACATCTCCATCACTTATCTCC-3′ | 936–957 | |
| | Oligonucleotide probe | 5′-CATCAGTGGCGATCTCCACATCCTG-3′ | 666–690 | |
| EGF | Sense primer | 5′-TGGTTGTGGTTCATCCATTGGC-3′ | 2628–2649 | 501 |
| | Antisense primer | 5′-GGCAGACATAACCACCTTCG-3′ | 3109–3128 | |
| | Oligonucleotide probe | 5′-GTTGATCTAAAGAACCAAGTAACAC-3′ | 2816–2846 | |
| TGF-α | Sense primer | 5′-GTAAAATGGTCCCCTCGG-3′ | 30–48 | 355 |
| | Antisense primer | 5′-GTGATGATAAGGACAGCCAGGG-3′ | 363–384 | |
| | Oligonucleotide probe | 5′-TAATGACTGCCCAGATTCCCACACT-3′ | 166–190 | |
| γ-Actin | Sense primer | 5′-CAAGTTCTACAATCCAGTGC-3′ | 395–414 | 474 |
| | Antisense primer | 5′-ACGAGACCACCTTCAACTCC-3′ | 849–868 | |

[a]Base pair positions are from sequences cited in the GenBank/EMBL data bank. Accession numbers for these sequences are as follows: EGF receptor, X00663; EGF, X04571; TGF-α, K03222; γ-actin, M16247.

### Results

### *Incidence of mRNA expression for EGF receptor, EGF and TGF-α in ovarian tumours*

All 97 ovarian tumours were analysed by RT–PCR for the presence of EGF receptor mRNA and 96 of these for the presence of EGF mRNA and TGF-α mRNA. An example of a typical RT–PCR gel is shown in Figure 1 and confirmation of the sequence of the transcript was obtained by use of a $^{32}$P-labelled probe, targeted to a unique sequence within the transcript (illustrated in Figure 1). Of the malignant tumours, 70% (53/76) were positive for EGF receptor mRNA, 31% (23/75) were positive for EGF mRNA and 35% (26/75) for TGF-α mRNA. Analysis of co-expression of receptor and ligand indicated that there was an association between the presence of mRNA for the EGF receptor and TGF-α mRNA ($P=0.0015$) but not between EGF receptor and EGF ($P=1.00$) (Table II). There was no association between the presence of mRNA for TGF-α and EGF ($P=1.00$) (Table II).

Of the six borderline tumours investigated, none expressed mRNA for EGF, while only one of six expressed TGF-α mRNA, however four of six expressed mRNA for EGF receptor. Fifteen benign tumours were also investigated, including seven of epithelial origin (five mucinous, one serous and one Brenner), three fibromas, one thecofibroma, one thecoma, two granulosa cell tumours and one mature cystic teratoma. Five of these 15 tumours contained EGF receptor mRNA. This was significantly different from the 70% incidence found for malignant tumours ($P=0.016$). Expression of EGF mRNA was found in 40% (6/15) benign tumours, which was similar to the level found in malignant tumours, while expression of TGF-α was observed in only 1/15 cases, significantly lower than the expression rate of 35% observed in the malignant group ($P=0.033$).

### *Relationship of mRNA expression to clinicopathological features*

The relationship between expression patterns and histology in malignant tumours is shown in Table III. EGF receptor mRNA expression was significantly more strongly associated with serous histology than other subtypes ($P=0.006$). In contrast, neither EGF nor TGF-α mRNA expression showed any association with histological subtype (Table III). None of these three mRNAs demonstrated any association with stage, amount of residual disease or grade of differentiation.

### *mRNA expression and prognosis*

The relationship between mRNA expression and survival was investigated in the malignant group overall (survival data available for 70 of the 76 patients) and in the epithelial malignant subgroup ($n=60$) (Table IV). For the malignant group overall, the presence of EGF receptor mRNA

**Figure 1** EGF receptor mRNA expression in primary ovarian tumours. (**a**) RT–PCR agarose gel wherein cDNA was reverse-transcribed from tumour RNA and amplified using primers shown in Table I. Lane 1 contains DNA molecular weight markers, lanes 2–7 contain DNA from ovarian tumours and lanes 3, 5 and 7 show a band at 516 consistent with EGF receptor amplified cDNA. Lane 8 is a negative control containing the PCR reaction mix in the absence of tumour. (**b**) Confirmation of EGF receptor transcript by Southern blot analysis using a specific 5′-$^{32}$P-labelled probe. Lanes 3, 5 and 7 correspond to the same lanes in the top panel and show the presence of a transcript.

Table II Relationships between EGF receptor, EGF and TGF-α mRNA expression

| | TGF-α expression Positive | TGF-α expression Negative | | EGF expression Positive | EGF expression Negative | |
|---|---|---|---|---|---|---|
| EGF receptor positive | 24 | 28 | $P=0.0015$ | 16 | 36 | $P=1.00$ |
| EGF receptor negative | 2 | 21 | | 7 | 16 | |
| TGF-α positive | | | | 8 | 18 | $P=1.00$ |
| TGF-α negative | | | | 15 | 34 | |

$P$ values obtained using Fisher's exact test.

Table III Relationship between mRNA expression and clinicopathological parameters

| Parameter | EGF receptor Positive | EGF receptor Negative | P-value | EGF Positive | EGF Negative | P-value | TGF-α Positive | TGF-α Negative | P-value |
|---|---|---|---|---|---|---|---|---|---|
| Histology | | | | | | | | | |
| Serous | 30 | 5 | | 9 | 26 | | 15 | 20 | |
| Endometrioid | 14 | 8 | 0.006[a] | 6 | 15 | 0.46[a] | 7 | 14 | 0.22[a] |
| Mucinous | 3 | 2 | | 1 | 4 | | 2 | 3 | |
| Clear cell | 1 | 4 | | 1 | 4 | | 1 | 4 | |
| MMMT | 3 | 0 | | 3 | 0 | | 0 | 3 | |
| Others | 2 | 4 | | 3 | 3 | | 1 | 5 | |
| Stage | | | | | | | | | |
| I/II | 14 | 11 | 0.054 | 10 | 15 | 0.43 | 5 | 20 | 0.072 |
| III/IV | 40 | 10 | | 14 | 35 | | 21 | 28 | |
| Residual disease | | | | | | | | | |
| <2 cm | 27 | 15 | 0.18 | 15 | 27 | 0.44 | 14 | 28 | 0.80 |
| >2 cm | 23 | 5 | | 7 | 20 | | 10 | 17 | |

[a]Comparison of serous *vs* rest (Fisher's exact test); for a few tumours, data on patient stage and amount of residual disease were not available; similarly for one of the endometrioid tumours, data were obtained for EGF receptor mRNA but not EGF or TGF-α mRNA.

expression was significantly associated with reduced survival ($P=0.030$), while there was no association between the presence of EGF ($P=0.33$) or TGF-α ($P=0.45$) and survival. Data on clinical parameters for this group of patients are also shown in Table IV. Of these, stage and amount of residual disease have the most significant effects on survival (for both, $P<0.001$).

Analysis of the patients with epithelial ovarian cancer (serous, endometrioid, mucinous and clear cell carcinomas) who make up almost 90% of the malignant group, indicated a more significant association of the EGF receptor mRNA with reduced survival ($P=0.007$) (Figure 2). As with the malignant group overall, the association between survival with EGF mRNA ($P=0.113$) and TGF-α ($P=0.119$) was non-significant, whereas stage, amount of residual disease, grade and histology were all linked to survival in this group of patients (Table IV). To assess if mRNA expression was independently prognostic for survival, Cox regression analysis was used. The hazard ratio for expression of EGF receptor mRNA alone in a Cox model is 3.9 (standard error = 2.0). Using only biological variables (i.e. omitting debulk), results for the Cox model are shown in Table V (62 patients, 33 events) and a hazard ratio of 2.5 for expression of EGF receptor mRNA was obtained. Using all variables, both debulk and EGF receptor mRNA expression remained in the final Cox model with the hazard ratio for EGF receptor being similar to that in the model above.

### Discussion

Expression of the EGF receptor is now widely accepted to confer poor prognosis in breast cancer patients, and evidence is accumulating that such a link may exist in other solid neoplasms including ovarian cancer (Bauknecht *et al.*, 1988; Battaglia *et al.*, 1989; Foekens *et al.*, 1990; Berchuck *et al.*, 1991; Scambia *et al.*, 1992). Novel treatment strategies targeted against the EGF receptor, are currently under investigation. Such treatments will, ultimately, require testing under conditions in patients known to be positive for the EGF receptor target molecule. The use of rapid and robust techniques for assessment of EGF receptor status is a prerequisite for successful targeting of such treatments. In this study, RT–PCR has been used to detect mRNA

Table IV Univariate analysis of survival

| Parameter | Comparison | P (log-rank) All malignant n = 70 | P (log-rank) Epithelial only n = 62 |
|---|---|---|---|
| EGF receptor mRNA | Positive *vs* negative | 0.030 | 0.007 |
| EGF mRNA | Positive *vs* negative | 0.33 | 0.11 |
| TGF-α mRNA | Positive *vs* negative | 0.45 | 0.12 |
| FIGO stage | Stage I *vs* stages II, III and IV | <0.001 | <0.001 |
| Residual disease | <2 cm *vs* >2 cm | <0.001 | <0.001 |
| Grade | Poor *vs* well + moderate | 0.008 | 0.003 |
| Histology | Serous *vs* rest | – | 0.039 |

**Figure 2** Relationship between EGF receptor expression and survival of patients with epithelial ovarian cancer. Survival of patients whose tumours expressed EGF receptor mRNA demonstrated reduced survival compared with patients whose tumours were negative ($P=0.007$; log-rank test).

expression in primary ovarian tumours. This approach has the advantages of sensitivity and avoids the problems of protein determination, such as masking of receptors by ligand or receptor internalisation. The only previous report of mRNA expression for EGF receptor and TGF-α in ovarian cancer does provide some evidence that mRNA levels correlate with protein levels (Kohler *et al.*, 1992). The aims of the current study were to expand the current database on EGF receptor in relation to disease outcome in ovarian cancer and to do so using rapid molecular biology techniques to assess receptor expression.

The data presented here, together with previously published studies, indicate that both the EGF receptor and its ligands are commonly present in ovarian tumours. Previous reports of the incidence of the EGF receptor protein in ovarian tumours vary between 33% and 75% when determined by ligand binding or immunohistochemical techniques (Bauknecht *et al.*, 1988, 1993; Battaglia *et al.*, 1989; Foekens *et al.*, 1990; Berchuck *et al.*, 1991; Morishige *et al.*, 1991; Owens *et al.*, 1991*a*; Berns *et al.*, 1992; Henzen-Logmans *et al.*, 1992). When mRNA for the EGF receptor was examined by Northern blot analysis, it was found to be present in 75% tumour samples (Kohler *et al.*, 1992; Bauknecht *et al.*, 1993). Our finding of 70% positivity for EGF receptor mRNA is therefore consistent with these reports. We found EGF mRNA present in 31% of ovarian tumours, which is again consistent with the finding of Owens and Leake (1993), who reported the protein to be present in 30% of malignant tumours. Our incidence of 35% positivity for TGF-α mRNA is lower than the 66% incidence for TGF-α mRNA determined by Northern blot analysis (Kohler *et al.*, 1992; Bauknecht *et al.*, 1993) or the 89% value reported for the protein by Owens *et al.* (1991*b*). This finding is despite the relative increase in sensitivity to be expected resulting from the use of RT–PCR in comparison with Northern blotting or protein estimation. The reason for such a discrepancy is unclear but it is unlikely to be related to methodological problems since all tumours assessed for mRNA expression of growth factors and receptors were first characterised for γ-actin mRNA expression as a control for integrity of extracted RNA. Furthermore, our ongoing studies with ovarian carcinoma cell lines have shown that expression of mRNA and protein for EGF, TGF-α and EGF receptor are in concordance, suggesting that this result is not due to an underestimation of TGF-α levels.

Investigation of a small group of 15 benign tumours indicated that 5 of 15 contained EGF receptor mRNA. While these numbers are small, they support the data of Owens and Leake (1993) who reported an incidence of only 13% EGF receptor protein positivity in benign ovarian tumours and do suggest that malignant tumours are more likely to express the EGF receptor than their benign counterparts.

A significant correlation was obtained between the presence of the EGF receptor mRNA and TGF-α mRNA in malignant tumours. This co-expression of EGF receptor with TGF-α but not with EGF in primary ovarian tumours was also noted by Morishige *et al.* (1991) and provides a basis for suggesting that a TGF-α/EGF receptor autocrine loop is present within ovarian cancer cells. This idea was supported by experiments using neutralising antibodies that produced growth inhibition in cultures obtained from ovarian primary tumours or ascites if the antibodies were targeted against TGF-α or the EGF receptor but not if targeted against EGF (Morishige *et al.*, 1991).

Investigation of EGF receptor mRNA expression rates in different ovarian tumour histological subtypes suggested a stronger association between EGF receptor mRNA and the most common and aggressive pathological subtype, the serous form. This finding is in agreement with the observation of Morishige *et al.* (1991) who, using ligand binding, found a higher incidence (74%-positive) of the EGF receptor in serous tumours when compared with other pathological subgroupings but contrasts with the majority of reports where no difference was identified (Bauknecht *et al.*, 1988; Owens *et al.*, 1991*a*; Scambia *et al.*, 1992; Van der Burg *et al.*, 1993). No association was found between the presence of EGF receptor mRNA and advanced stage in agreement with previous studies (Bauknecht *et al.*, 1988; Berchuck *et al.*, 1991; Owens *et al.*, 1991*a*; Van der Burg *et al.*, 1993). However, some studies (Battaglia *et al.*, 1989; Henzen-Logmans *et al.*, 1992) have reported an increased incidence of positivity in metastatic lesions as compared with primary tumours which, in turn, might suggest increased expression is associated with disease progression. Perhaps the strongest indication that the EGF receptor could play a role in the course of this disease is its proposed link with prognosis. Our findings support the view that the presence of EGF receptor mRNA is associated with reduced survival in malignant tumours when examined by univariate analysis. Furthermore this relationship appears to be stronger within the epithelial group, which represents 89% of this series of malignant tumours. The hazard ratio for EGF receptor mRNA alone in a Cox model is 3.9. Multivariate analysis however demonstrated that this was not significantly independent ($P=0.1$) of the clinicopathological parameters studied. Four groups have published on the relationship of the EGF receptor protein with respect to prognosis. Berchuck *et al.* (1991), using immunohistochemical detection, demonstrated that the presence of EGF receptor was associated with reduced overall survival. Scambia *et al.* (1992), using a ligand binding technique, demonstrated that progression-free survival was reduced in patients with EGF receptor-positive tumours, again suggesting that EGF receptor positivity is associated with a negative outcome. Other reports however have contradicted these findings. Van der Burg *et al.* (1993)

**Table V** Multivariate analysis of survival in epithelial cancer patients

| *Variable* | *Classification* | *Hazard ratio* | *95% CI*[a] | P-*value* |
|---|---|---|---|---|
| EGF receptor | Positive *vs* negative | 2.5 | (0.8, 7.2) | 0.1 |
| Grade | Poor *vs* well + moderate | 2.2 | (1.0, 5.1) | 0.06 |
| Stage | I *vs* II-IV | 3.5 | (1.0, 12.1) | 0.05 |

[a]CI, confidence interval.

found no correlation between the presence of EGF receptor protein, determined either by immunohistochemistry or ligand binding, and progression-free survival. Furthermore, initial studies by Bauknecht *et al.* suggested that patients with EGF receptor-positive tumours had improved overall survival (Bauknecht *et al.*, 1988), however extension of this data set to include more patients reversed this finding (Bauknecht *et al.*, 1990). In a subsequent report, the same authors found that ovarian tumours expressing high concentrations of EGF receptor and TGF-α responded better initially to chemotherapy than those with negative or low levels of these proteins, however recurrence was also rapid in the same group (Bauknecht *et al.*, 1993).

In conclusion, this is the first report on the use of RT–PCR methodology to determine expression of the EGF family in ovarian cancer. Expression of EGF receptor mRNA was found to be associated with serous histology and a poor survival. These data support the view that expression of the EGF receptor plays a key role in the biology of this disease.

**Acknowledgements**

D Katsaros is an AIRC (Associazione Italiana per la Ricerca sul cancro) fellowship recipient.

### References

BARTLETT JMS, RABIASZ GM, SCOTT WN, LANGDON SP, SMYTH JF AND MILLER WR. (1992). Transforming growth factor-β mRNA expression and growth control of human ovarian carcinoma cells. *Br. J. Cancer*, **65**, 655–660.

BATTAGLIA F, SCAMBIA G, BENEDITTI PANICI P, BAIOCCHI G, PERRONI L, IACOBELLI S AND NANCUROS S. (1989). Epidermal growth factor expression in gynecological malignancies. *Gynecol. Obstet. Invest.*, **27**, 42–44.

BAUKNECHT T, RUNGE M, SCHWALL M AND PFLEIDERER A. (1988). Occurrence of epidermal growth factor receptors in human adnexal tumors and their prognostic value in advanced ovarian carcinomas. *Gynecol. Oncol.*, **29**, 147–157.

BAUKNECHT T, BIRMELIN G AND KOMMOSS F. (1990). Clinical significance of oncogenes and growth factors in ovarian carcinomas. *J. Ster. Biochem. Mol. Biol.*, **37**, 855–862.

BAUKNECHT T, ANGEL P, KOHLER M, KOMMOSS F, BIRMELIN G, PFLEIDERER A AND WAGNER E. (1993). Gene structure and expression analysis of the epidermal growth factor receptor, transforming growth factor alpha, myc, jun and metallothionein in human ovarian carcinomas: classification of malignant phenotypes. *Cancer*, **71**, 419–429.

BERCHUCK A, RODRIGUEZ GC, KAMEL A, DODGE RK, SOPER JT, CLARKE-PEARSON DL AND BAST RC. (1991). Epidermal growth factor expression in normal ovarian epithelium and ovarian cancer. *Am. J. Obstet. Gynecol.*, **164**, 669–674.

BERCHUCK A AND BAST RC JR. (1993). Growth factors, oncogenes and tumor suppressor genes. In *Cancer of the Ovary*. Markman M and Hoskins WJ (eds) pp. 61–78. Raven Press: New York.

BERNS EMJJ, KLIJN JGM, HENZEN-LOGMANS SC, RODENBURG CJ AND VAN DER BURG MEL. (1992). Receptors for hormones and growth factors and (onco)-gene amplification in human ovarian cancer. *Int. J. Cancer*, **52**, 218–224.

CANNISTRA SA. (1993). Cancer of the ovary. *N. Engl. J. Med.*, **329**, 1550–1559.

COX DR. (1972). Regression models and life tables. *J. R. Stat. Soc. (B)*, **34**, 187–220.

CREW AJ, LANGDON SP, MILLER EP AND MILLER WR. (1992). Mitogenic effects of epidermal growth factor and transforming growth factor-α on EGF-receptor positive human ovarian carcinoma cell lines. *Eur. J. Cancer*, **28**, 337–341.

FOEKENS JA, VAN-PUTTEN WL, PORTENGEN H, RODENBURG CJ, REUBI JC, BERNS PM, HENZEN-LOGMANS SC, VAN DER BURG ME, ALEXIEVA-FIGUSH J AND KLIJN JG. (1990). Prognostic value of pS2 protein and receptors for epidermal growth factor (EGF-R), insulin-like growth factor 1 (IGF-1-R) and somatostatin in patients with breast and ovarian cancer. *J. Ster. Biochem. Mol. Biol.*, **37**, 815–821.

HENZEN-LOGMANS SC, BERNS EMJJ, KLIJN JGM, VAN DER BURG MEL AND FOEKENS JA. (1992). Epidermal growth factor receptor in ovarian tumours: correlation of immunohistochemistry with ligand binding assay. *Br. J. Cancer*, **66**, 1015–1021.

INTERNATIONAL FEDERATION OF GYNECOLOGY AND OBSTETRICS. (1987). Changes in definitions of clinical staging for carcinoma of the cervix and ovary. *Am. J. Obstet. Gynecol.*, **156**, 263–264.

JINDAL SK, SNOEY DM, LOBB DK AND DORRINGTON JH. (1994). Transforming growth factor alpha localization and role in surface epithelium of normal human ovaries and in ovarian carcinoma cells. *Gynecol. Oncol.*, **53**, 17–23.

KOHLER M, BAUKNECHT T, GRIMM M, BIRMELIN G, KOMMOSS F AND WAGNER E. (1992). Epidermal growth factor receptor (EGF-R) and transforming growth factor alpha (TGF-α) expression in human ovarian carcinomas. *Eur. J. Cancer*, **8–9**, 1432–1437.

LA VECCHIA C, LUCCHINI F, NEGRI E, BOYLE P, MAISONNEUVE P AND LEVI F. (1992). Trends of cancer mortality in Europe, 1955–1989: III, breast and genital sites. *Eur. J. Cancer*, **28A**, 927–998.

MORISHIGE K, KURACHI H, AMEMIYA K, FUJITA Y, YAMAMOTO T, MIYAKE A AND TANIZAWA O. (1991). Evidence for the involvement of transforming growth factor-α and epidermal growth factor receptor autocrine growth mechanism in primary human ovarian cancers in vitro. *Cancer Res.*, **51**, 5322–5328.

OWENS OJ AND LEAKE RE. (1993). Epidermal growth factor receptor expression in malignant ovary, benign ovarian tumours and normal ovary: a comparison. *Int. J. Cancer*, **2**, 321–324.

OWENS OJ, STEWART C, BROWN I AND LEAKE RE. (1991). Epidermal growth factor receptors (EGFR) in human ovarian cancer. *Br. J. Cancer*, **64**, 907–910.

OWENS OJ, STEWART C AND LEAKE RE. (1991). Growth factors in ovarian cancer. *Br. J. Cancer*, **64**, 1177–1181.

OWENS OJ, STEWART C, LEAKE RE AND MCNICOL AM. (1992). A comparison of biochemical and immunohistochemical assessment of EGFr expression in ovarian cancer. *Anticancer Res.*, **12**, 1455–1458.

RODRIGUEZ GC, BERCHUCK A, WHITAKER RS, SCHLOSSMAN D, CLARKE-PEARSON DL AND BAST RC. (1991). Epidermal growth factor receptor expression in normal ovarian epithelium and ovarian cancer. 2. Relationship between receptor expression and response to epidermal growth factor. *Am. J. Obstet. Gynecol.*, **164**, 745–750.

SCAMBIA G, BENEDETTI-PANICI P, BATTAGLIA F, FERRANDINA G, GAGGINI C AND MANCUSO S. (1991). Presence of epidermal growth factor (EGF) receptor and proliferative response to EGF in six human ovarian carcinoma cell lines. *Int. J. Gynecol. Cancer*, **1**, 253–258.

SCAMBIA G, BENEDETTI-PANICI P, BATTAGLIA F, FERRANDINA G, BAIOCCHI G, GREGGI S, DE VINCENZO R AND MANCUSO S. (1992). Significance of epidermal growth factor receptor in advanced ovarian cancer. *J. Clin. Oncol.*, **10**, 529–535.

SEROV S AND SCULLY RE. (1973). Histological typing of ovarian tumors. In *International Histological Classification of Tumors*, no 9, World Health Organization: Geneva.

VAN DER BURG MEL, HENZEN-LOGMANS SC, FOEKENS JA, BERNS EMJJ, RODENBURG CJ, VAN PUTTEN WLJ AND KLIJN JGM. (1993). The prognostic value of epidermal growth factor receptors, determined by both immunohistochemistry and ligand binding assays, in primary epithelial ovarian cancer: a pilot study. *Eur. J. Cancer*, **29A**, 1951–1957.

ZHOU L AND LEUNG BS. (1992). Growth regulation of ovarian cancer cells by epidermal growth factor and transforming growth factors α and β1. *Biochim. Biophys. Acta*, **1180**, 130–136.


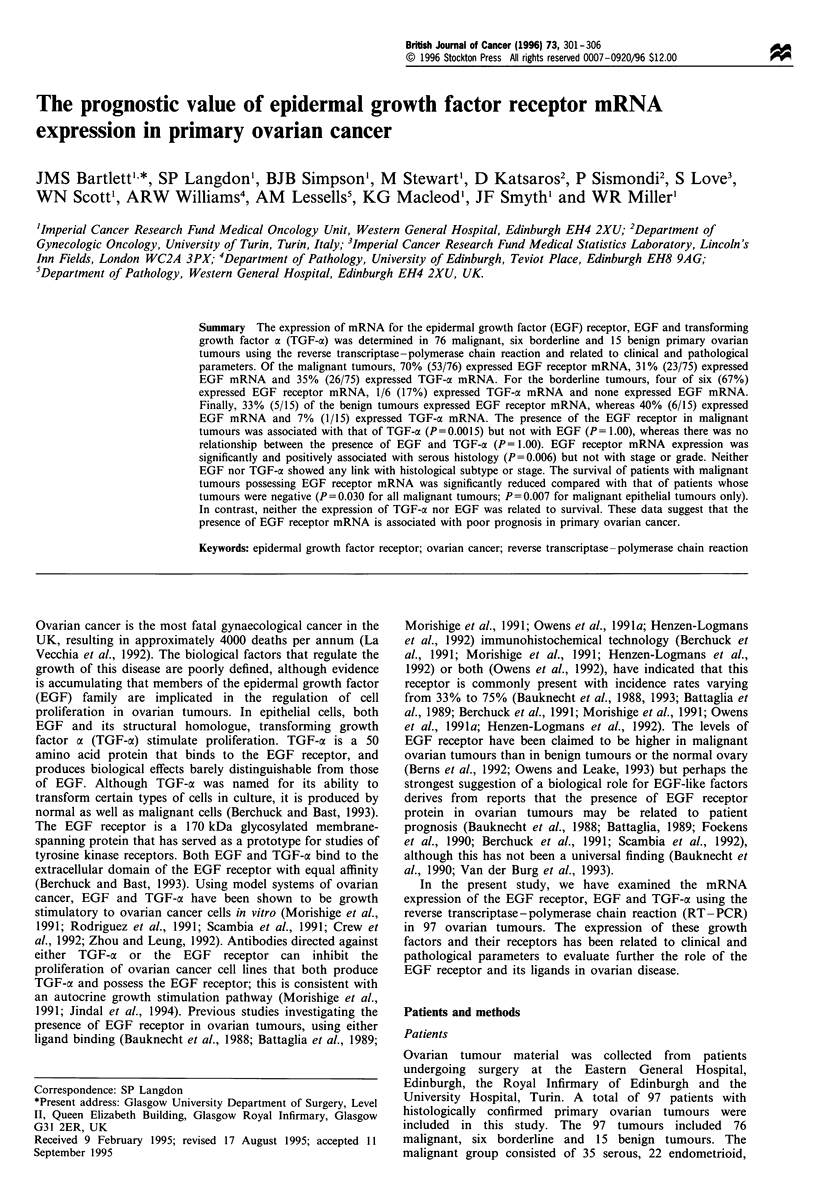

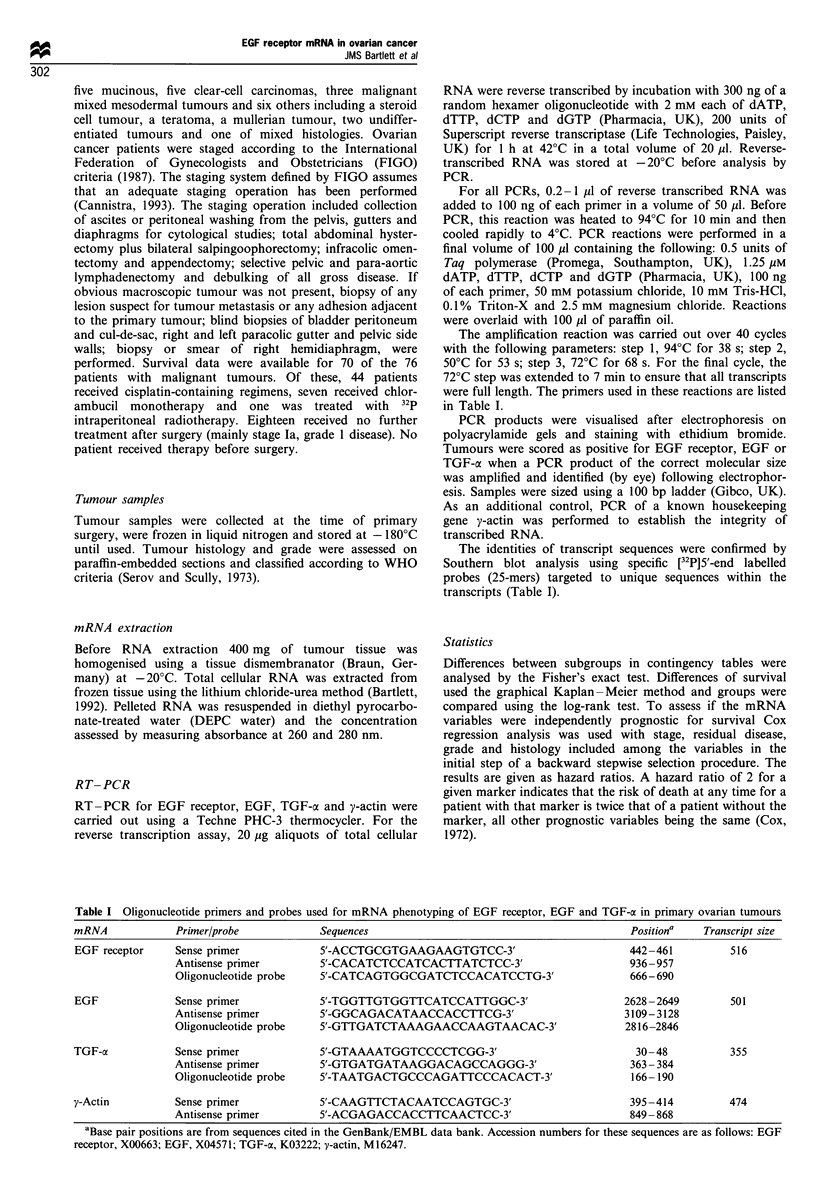

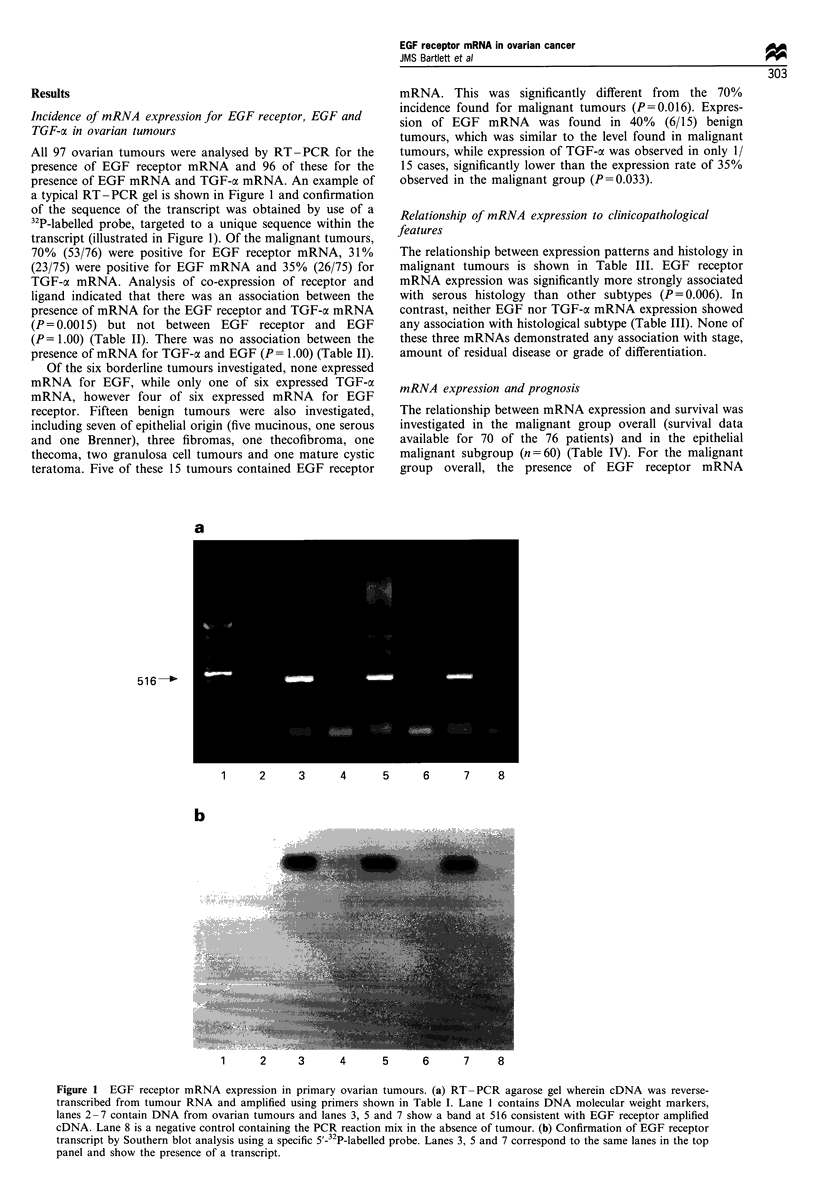

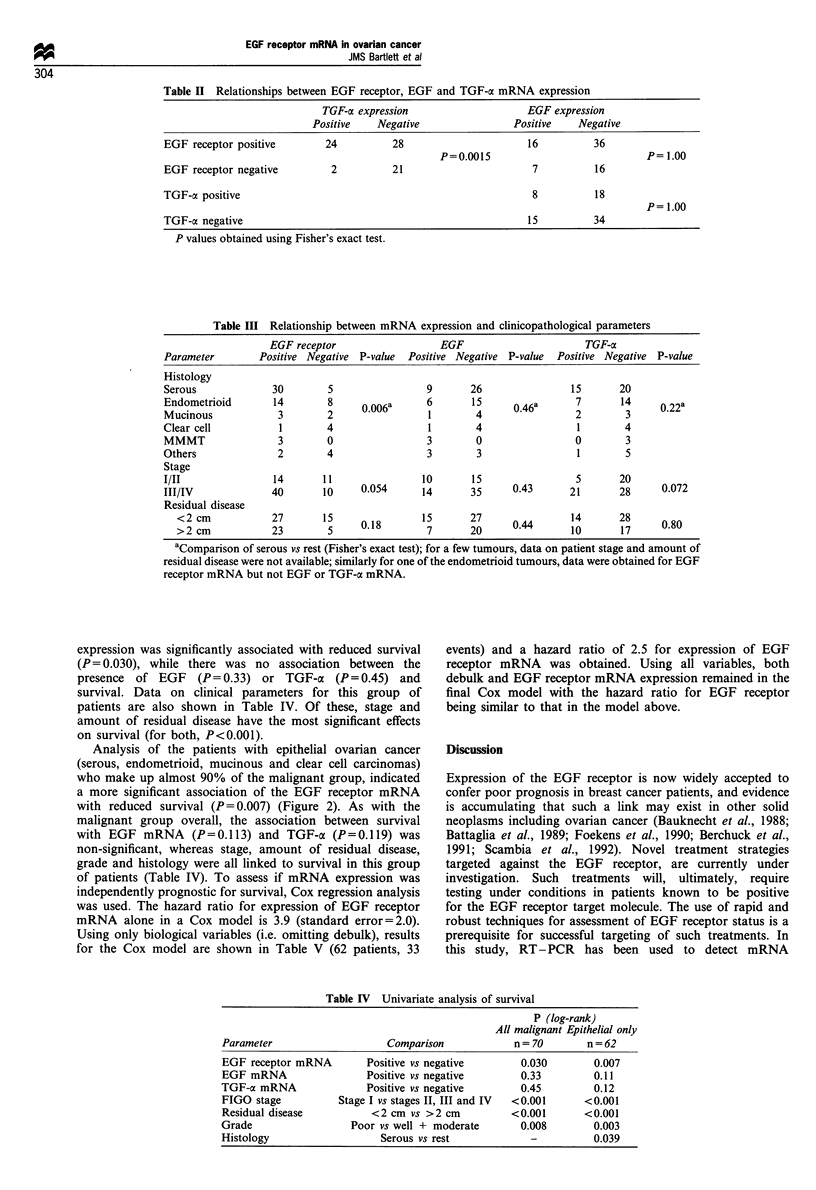

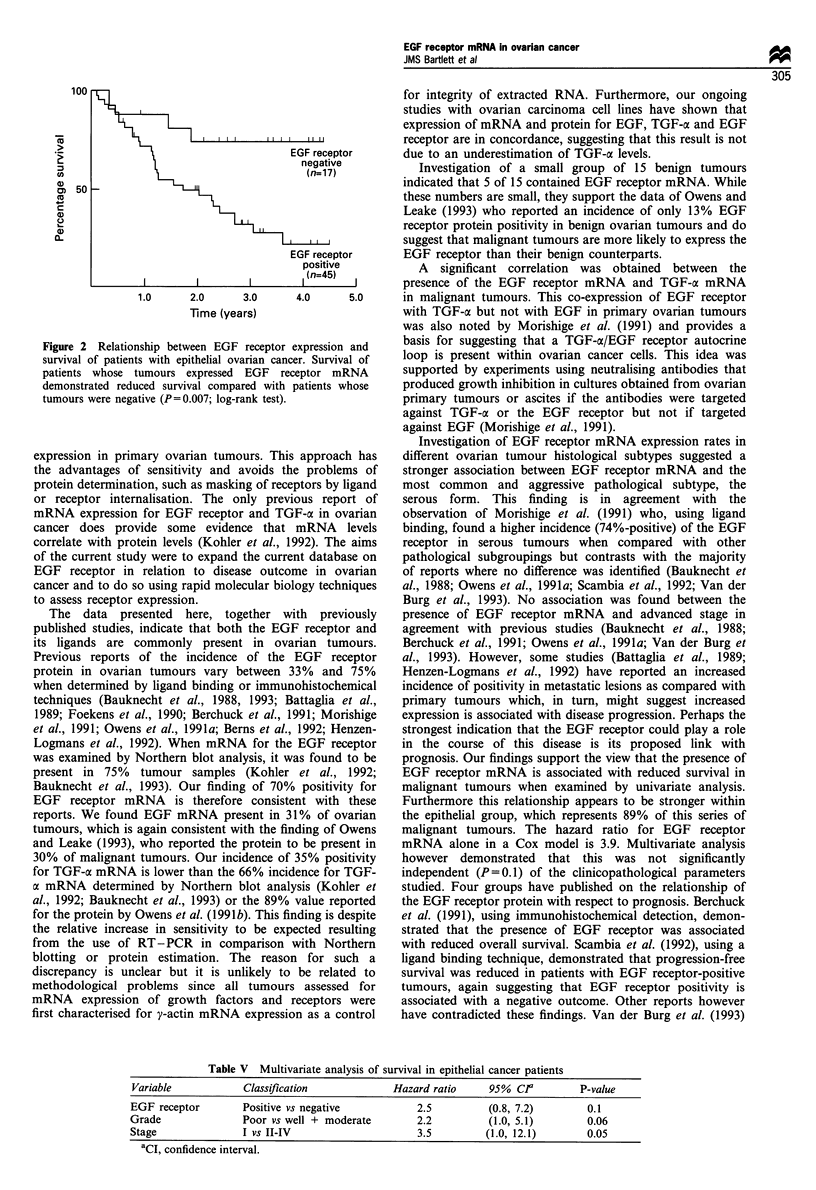

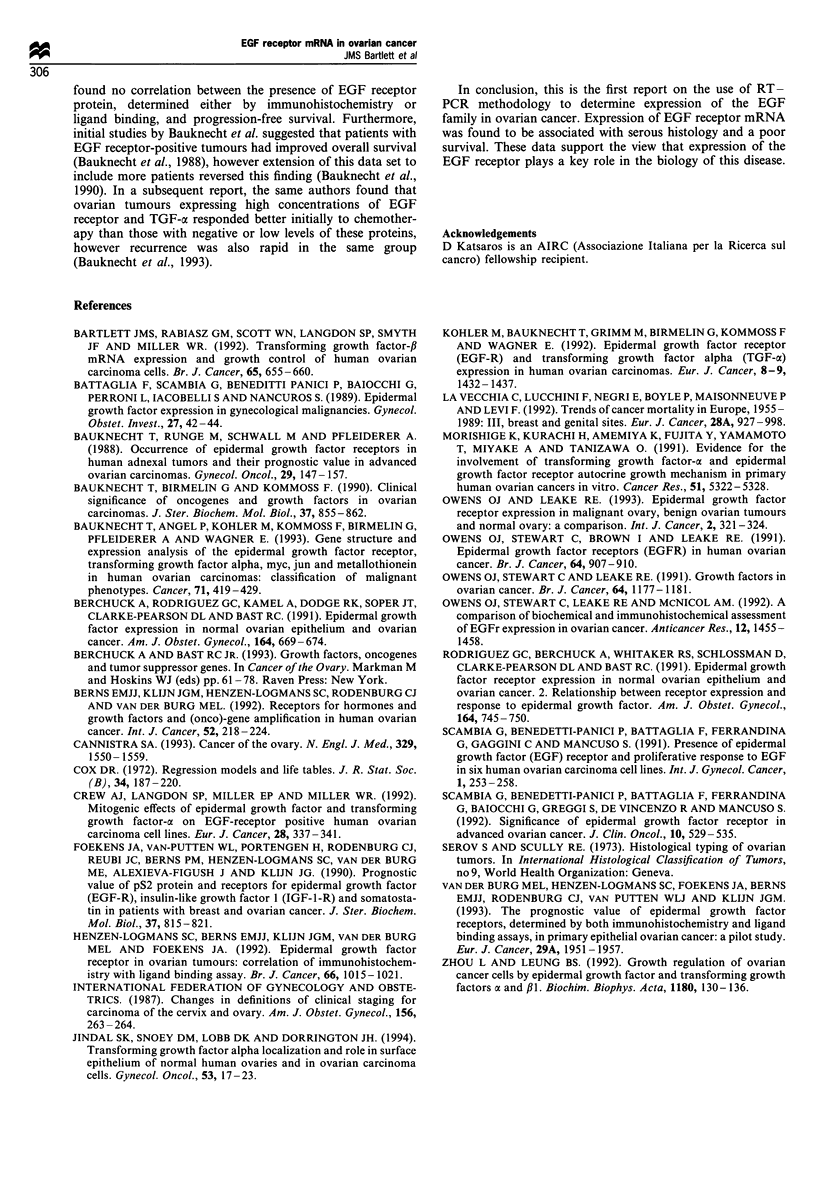


## References

[OCR_00634] Bartlett J. M., Rabiasz G. J., Scott W. N., Langdon S. P., Smyth J. F., Miller W. R. (1992). Transforming growth factor-beta mRNA expression and growth control of human ovarian carcinoma cells.. Br J Cancer.

[OCR_00641] Battaglia F., Scambia G., Benedetti Panici P., Baiocchi G., Perrone L., Iacobelli S., Mancuso S. (1989). Epidermal growth factor receptor expression in gynecological malignancies.. Gynecol Obstet Invest.

[OCR_00655] Bauknecht T., Angel P., Kohler M., Kommoss F., Birmelin G., Pfleiderer A., Wagner E. (1993). Gene structure and expression analysis of the epidermal growth factor receptor, transforming growth factor-alpha, myc, jun, and metallothionein in human ovarian carcinomas. Classification of malignant phenotypes.. Cancer.

[OCR_00652] Bauknecht T., Birmelin G., Kommoss F. (1990). Clinical significance of oncogenes and growth factors in ovarian carcinomas.. J Steroid Biochem Mol Biol.

[OCR_00646] Bauknecht T., Runge M., Schwall M., Pfleiderer A. (1988). Occurrence of epidermal growth factor receptors in human adnexal tumors and their prognostic value in advanced ovarian carcinomas.. Gynecol Oncol.

[OCR_00666] Berchuck A., Rodriguez G. C., Kamel A., Dodge R. K., Soper J. T., Clarke-Pearson D. L., Bast R. C. (1991). Epidermal growth factor receptor expression in normal ovarian epithelium and ovarian cancer. I. Correlation of receptor expression with prognostic factors in patients with ovarian cancer.. Am J Obstet Gynecol.

[OCR_00674] Berns E. M., Klijn J. G., Henzen-Logmans S. C., Rodenburg C. J., van der Burg M. E., Foekens J. A. (1992). Receptors for hormones and growth factors and (onco)-gene amplification in human ovarian cancer.. Int J Cancer.

[OCR_00680] Cannistra S. A. (1993). Cancer of the ovary.. N Engl J Med.

[OCR_00690] Crew A. J., Langdon S. P., Miller E. P., Miller W. R. (1992). Mitogenic effects of epidermal growth factor and transforming growth factor-alpha on EGF-receptor positive human ovarian carcinoma cell lines.. Eur J Cancer.

[OCR_00698] Foekens J. A., van Putten W. L., Portengen H., Rodenburg C. J., Reubi J. C., Berns P. M., Henzen-Logmans S. C., van der Burg M. E., Alexieva-Figusch J., Klijn J. G. (1990). Prognostic value of pS2 protein and receptors for epidermal growth factor (EGF-R), insulin-like growth factor-1 (IGF-1-R) and somatostatin (SS-R) in patients with breast and ovarian cancer.. J Steroid Biochem Mol Biol.

[OCR_00705] Henzen-Logmans S. C., Berns E. M., Klijn J. G., van der Burg M. E., Foekens J. A. (1992). Epidermal growth factor receptor in ovarian tumours: correlation of immunohistochemistry with ligand binding assay.. Br J Cancer.

[OCR_00717] Jindal S. K., Snoey D. M., Lobb D. K., Dorrington J. H. (1994). Transforming growth factor alpha localization and role in surface epithelium of normal human ovaries and in ovarian carcinoma cells.. Gynecol Oncol.

[OCR_00723] Kohler M., Bauknecht T., Grimm M., Birmelin G., Kommoss F., Wagner E. (1992). Epidermal growth factor receptor and transforming growth factor alpha expression in human ovarian carcinomas.. Eur J Cancer.

[OCR_00728] La Vecchia C., Lucchini F., Negri E., Boyle P., Maisonneuve P., Levi F. (1992). Trends of cancer mortality in Europe, 1955-1989: III, Breast and genital sites.. Eur J Cancer.

[OCR_00735] Morishige K., Kurachi H., Amemiya K., Fujita Y., Yamamoto T., Miyake A., Tanizawa O. (1991). Evidence for the involvement of transforming growth factor alpha and epidermal growth factor receptor autocrine growth mechanism in primary human ovarian cancers in vitro.. Cancer Res.

[OCR_00746] Owens O. J., Stewart C., Brown I., Leake R. E. (1991). Epidermal growth factor receptors (EGFR) in human ovarian cancer.. Br J Cancer.

[OCR_00751] Owens O. J., Stewart C., Leake R. E. (1991). Growth factors in ovarian cancer.. Br J Cancer.

[OCR_00753] Owens O. J., Stewart C., Leake R. E., McNicol A. M. (1992). A comparison of biochemical and immunohistochemical assessment of EGFR expression in ovarian cancer.. Anticancer Res.

[OCR_00762] Rodriguez G. C., Berchuck A., Whitaker R. S., Schlossman D., Clarke-Pearson D. L., Bast R. C. (1991). Epidermal growth factor receptor expression in normal ovarian epithelium and ovarian cancer. II. Relationship between receptor expression and response to epidermal growth factor.. Am J Obstet Gynecol.

[OCR_00777] Scambia G., Benedetti Panici P., Battaglia F., Ferrandina G., Baiocchi G., Greggi S., De Vincenzo R., Mancuso S. (1992). Significance of epidermal growth factor receptor in advanced ovarian cancer.. J Clin Oncol.

[OCR_00769] Scambia G., Ranelletti F. O., Benedetti Panici P., Piantelli M., Bonanno G., De Vincenzo R., Ferrandina G., Pierelli L., Capelli A., Mancuso S. (1991). Quercetin inhibits the growth of a multidrug-resistant estrogen-receptor-negative MCF-7 human breast-cancer cell line expressing type II estrogen-binding sites.. Cancer Chemother Pharmacol.

[OCR_00793] Zhou L., Leung B. S. (1992). Growth regulation of ovarian cancer cells by epidermal growth factor and transforming growth factors alpha and beta 1.. Biochim Biophys Acta.

[OCR_00785] van der Burg M. E., Henzen-Logmans S. C., Foekens J. A., Berns E. M., Rodenburg C. J., van Putten W. L., Klijn J. G. (1993). The prognostic value of epidermal growth factor receptors, determined by both immunohistochemistry and ligand binding assays, in primary epithelial ovarian cancer: a pilot study.. Eur J Cancer.

